# Sexual dysfunction in female patients with relapsing‐remitting multiple sclerosis

**DOI:** 10.1002/brb3.699

**Published:** 2017-04-14

**Authors:** Pawel Bartnik, Aleksandra Wielgoś, Joanna Kacperczyk, Katarzyna Pisarz, Iwona Szymusik, Aleksandra Podlecka‐Piętowska, Beata Zakrzewska‐Pniewska, Miroslaw Wielgoś

**Affiliations:** ^1^ Students’ Research Group at the 1st Department of Obstetrics and GynecologyMedical University of WarsawWarsawPoland; ^2^ Department of Experimental and Clinical PhysiologyMedical University of WarsawWarsawPoland; ^3^ Students’ Research Group at the Department of NeurologyMedical University of WarsawWarsawPoland; ^4^ 1st Department of Obstetrics and GynecologyMedical University of WarsawWarsawPoland; ^5^ Department of NeurologyMedical University of WarsawWarsawPoland

**Keywords:** depression, multiple sclerosis, neuroepidemiology, neuropsychology

## Abstract

**Introduction:**

Sexual dysfunction (SD) is one of the common symptoms of multiple sclerosis (MS) and is often underdiagnosed, especially in women. Relapsing‐remitting multiple sclerosis (RRMS) is the most widespread form of the disease, but the data on SD occurrence in this particular group of patients is limited. The aim of the study was to analyze the associations between demographic factors, symptoms and signs of MS, psychiatric comorbidities and SD in female patients with RRMS.

**Material & Methods:**

A subgroup of 86 sexually active women with RRMS out of 218 total MS respondents was analyzed. Exclusion criteria included active relapse, EDSS score equal or higher than 6.5, and current pregnancy. All patients completed questionnaires including demographic data, questions about symptoms and signs of MS, Female Sexual Function Index (FSFI) for sexual performance, Patient Health Questionnaire 9 (PHQ‐9) for depression, and Fatigue Severity Scale (FSS) for fatigue evaluation.

**Results:**

According to FSFI, SD occurred in 21 (27.27%) of the respondents. SD occurrence was associated with depression (*p* < .05) and speech disturbances (*p* < .04). A negative effect on sexual performance was associated with depression intensity (*p* < .003), fatigue intensity (*p* < .05), more advanced age at diagnosis (*p* < .02), lower education level (*p* < .05), and smaller area of residence (*p* < .002).

**Conclusions:**

SD in women with RRMS is mostly associated with psychosocial parameters. Patients who are more depressed, presenting speech problems, less educated, and from smaller towns, should be considered high‐risk for sexual dysfunction.

## 1 Introduction

Multiple sclerosis (MS) is a chronic, inflammatory, demyelinating, and neurodegenerative disease with a typical onset between 3rd and 4th decade of life (Goodin, 2014). It affects mostly women, with increasing women to men ratio (Koch‐Henriksen & Sorensen, 2010). The most common form of MS is the relapsing‐remitting MS (RRMS), which has an onset at a younger age when compared to other forms of this disease (Goodin, 2014).

Sexual dysfunction (SD) is considered to be one of the most widespread symptoms of the disease, affecting 50%–83% of the female patients with MS, depending on study (Barak et al., 1996; Lew‐Starowicz & Rola, 2013; Zorzon et al., 1999). SD has a significant impact on the quality of life of patients with MS (Nortvendt et al., 2001). These dysfunctions are also often underdiagnosed among women, but affect them more frequently than men (Celik et al., 2013; Zorzon et al., 1999).

Sexual dysfunctions in patients with MS are often divided into primary—caused by direct demyelination in regions affecting sexual response, resulting in inability to achieve orgasm, difficulties with arousal or genital sensation, secondary—caused by physical signs associated with sexual response, such as increased fatigue, pain due to inadequate lubrication, motor deficit or muscle spasticity, and tertiary—caused by psychological and sociological impairment, which often leads to lower self‐esteem and depression which have a negative effect on sexuality (Previnaire, Lecourt, Soler, & Denys, 2014).

Data about factors associated with their occurrence are limited and it is often hard to draw practical conclusions, as the MS population is extremely heterogeneous and most studies analyze general MS population as a whole (Darija et al., 2015; Fraser, Mahoney, & McGurl, 2008; Ghajarzedeh, Jalilian, Mohammadifar, Sahrain, & Azimi, 2014; Lew‐Starowicz & Rola, 2013; Merghati‐Khoei, Qaderi, Amini, & Korte, 2013; Mohammadi, Rahnama, Mohseni, Sahraian, & Montazeri, 2013; Zivadinov et al., 1999).

The primary aim of the study was to analyze the associations between sexual dysfunction occurrence and demographic factors, symptoms and signs of MS, psychiatric comorbidities among female patients with RRMS. The secondary objectives included the analysis of the associations between factors mentioned above and sexual performance of female patients with RRMS, measured by general Female Sexual Function Index (FSFI) score along with its specific subscales.

## 2 Material and Methods

**Figure 1:**
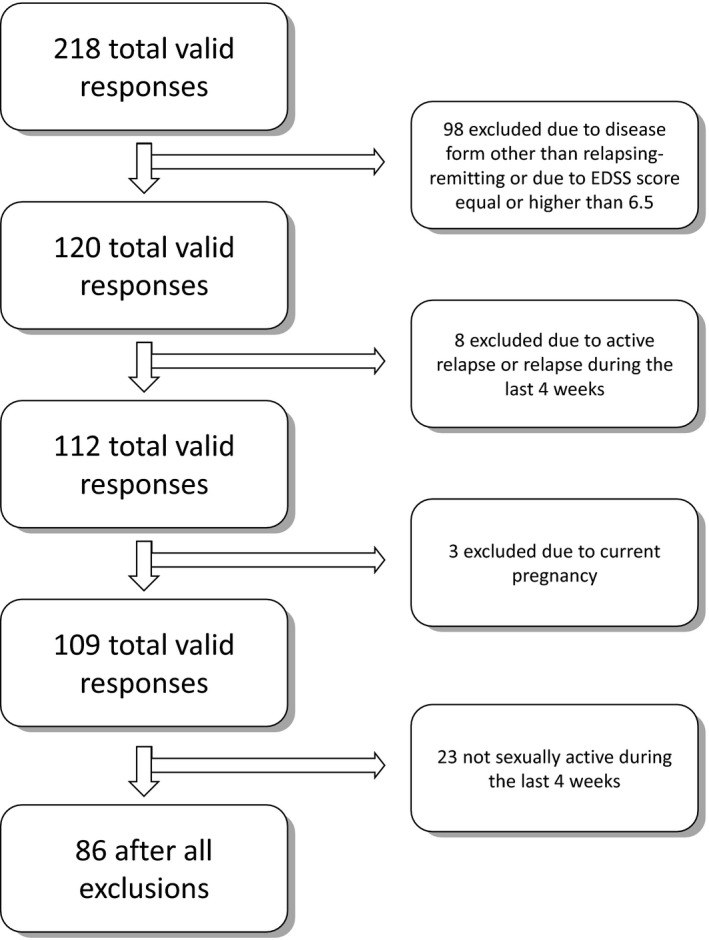
Study population profile

It was a cross‐sectional survey‐based study on selected 86 female, sexually active patients with RRMS. The initial group included 218 respondents with various forms and stages of MS, however, strict exclusion criteria were applied as presented in Figure 1. The exclusion criteria were: form of the disease other than RRMS or unknown form, current pregnancy, active relapse or relapse during 30 days prior to survey due to significant changes in all life activities during this period, including sexuality, and advanced stages of MS defined as Expanded Disability Status Scale (EDSS) score equal as or higher than 6.5. The form of the disease was self‐reported, with a strong indication in the question to answer “form uncertain” if the respondent is not sure about her form of the disease.

The survey was distributed among potential respondents using three routes. First one was an official mean of the Polish Society of Multiple Sclerosis. Female members of the Society received an invitation to an online, anonymous survey. The second group of respondents consisted of female patients of the Foundation of Urszula Jaworska, who also completed the questionnaires online and the last group were the patients of the Department of Neurology, Medical University of Warsaw. The proportion of valid surveys obtained from the mentioned sources is as follows (before and after application of exclusion criteria, respectively): the Polish Society of Multiple Sclerosis—119 and 54; Urszula Jaworska Foundation—70 and 25; Department of Neurology—29 and 7.

It is important to mention that only patients who were sexually active 4 weeks prior to survey were included in the final, analyzed group. It was due to the fact that the reason for lack of sexual activity could not be estimated without detailed history. The lack of sexual activity could either be a result of severe SD or come from other, non‐pathological causes. In order not to create unnecessary bias those respondents were not taken into analysis.

All of the respondents received a set of questionnaires, which consisted of modules, concerning the following issues: demographic data, symptoms, signs and course of MS, sexual performance, including full Female Sexual Function Index scale, reproductive health including pregnancy status, menopausal status and psychological comorbidities, including Patient Health Questionnaire 9 (PHQ‐9) scale for depression evaluation and Fatigue Severity Scale (FSS) for fatigue evaluation (Kroenke, Spitzer, & Williams, 2001; Krupp, LaRocca, Muir‐Nash, & Steinberg, 1989; Rosen et al., 2000).

The severity of symptoms and signs of MS was mostly evaluated subjectively by the respondents. Questions were designed and modeled on the example of two questions from the Urinary Bothersome Questionnaire for Multiple Sclerosis (Amarenco et al., 2013). The severity of subjective symptoms and signs had four stages: no sign/symptom present, mild, moderate, and severe.

The FSFI questionnaire consisted of questions concerning the following aspects of sexuality: desire, arousal, orgasm, lubrication, satisfaction, and pain.

The presence of Sexual Dysfunction (SD) was defined as achieving a score of 26.55 or lower in FSFI Questionnaire (only if all questions had been answered). The presence of depression was defined as achieving a score of 20 or more in PHQ‐9 Questionnaire and the presence of severe fatigue as achieving a score of 36 or above in FSS Questionnaire. The survey was self‐reported and fully anonymous in order to ensure the comfort of response.

Statistical analysis was performed using Statistica 12 (StatSoft. Inc.). U‐Mann Whitney test and *t*‐student tests were used for quantitative data comparison between two groups and Kruskall‐Wallis ANOVA test was applied for the comparison of three or more groups including quantitative data. Two‐sided Fisher's exact test was used for categorical and binary data comparison. *p* value <.05 was considered significant.

## 3 Results

The group characteristics are presented in Table 1. The vast majority of respondents was premenopausal (97.67%) and was receiving some form of disease‐modifying treatment (81.40%). Sexual dysfunction was revealed in 21 (27.27%) of the respondents.

**Table 1:** Group characteristics

Continuous variables				
Feature	Average (±*SD*)	Median	Minimum	Maximum
Age (years)	32.03 ± 7.22	31	20	51
Age at diagnosis (years)	24.25 ± 6.57	23	14	44
Disease duration (years)	7.87 ± 5.38	6	1	30
EDSS^a^	2.03 ± 1.44	2	0	6
Dichotomous variables				
Feature				
Ongoing disease‐modifying therapy (%)	70 (81.40)			
Post‐menopausal (%)	2 (2.33)			
Severe depression (%)	3 (3.57)			
Severe fatigue (%)	51 (62.96)			

Categorical variables				
	Rural	Small town (<20.000)	Town (20.000–100.000)	Town >100.000
Area of residence	16 (13.33)	18 (15.00)	24 (20.00)	62 (51.67)
Education	0	29 (24.17)	3 (2.5)	79 (63.83)
Marital status	51 (42.50)	57 (47.50)	3 (2.50)	9 (7.50)
Occupation	78 (65.00)	5 (4.17)	23 (19.17)	14 (11.17)

a Total *n* in this case‐45.

Table 2 presents the analysis of associations between demographic factors, self‐reported EDDS score, PHQ‐9, FSS scores, and the SD occurrence. The group with SD had a statistically higher PHQ‐9 score (11.33 vs. 8.07; *p* < .05), higher proportion of respondents with low education level (38.10% vs. 14.29%; *p* < .03), and higher proportion of residence in rural area or towns of less than 10.000 inhabitants (52.38% vs. 21.43%; *p* < .01).

**Table 2:** Continuous variables depending on SD presence

Feature	Sexual dysfunction present	No sexual dysfunction present	*p*
*N* (%)	21 (27.27)	56 (72.73)	‐
Age	32.90 ± 9.73	31.45 ± 6.26	.96
Low education level (%)	8 (38.10)	8 (14.29)	.03
Rural or town <10.000 residence (%)	11 (52.38)	12 (21.43)	.01
Unemployed (%)	5 (23.81)	9 (16.07)	.51
Not married (%)	12 (57.14)	32 (57.14)	1.00
Age at diagnosis	26.85 ± 8.31	23.28 ± 5.97	.10
Disease duration	5.84 ± 4.19	8.33 ± 5.64	.09
Self‐reported EDSS score^a^	2.06 ± 1.45	2.21 ± 1.57	.81
PHQ‐9 score	11.43 ± 6.83	8.07 ± 5.29	.05
FSS score	41.30 ± 14.54	40.04 ± 13.69	.87

a Total *n* in this case‐26.

FSS, fatigue severity scale, PHQ, patient health questionnaire.

Table 3 presents the analysis of associations between various MS symptoms and signs and SD occurrence. Among all of the presented signs only the occurrence of speech disturbances was associated with SD (47.37% vs. 21.05%; *p* < .04).

**Table 3:** SD occurrence depending on severity of MS signs and symptoms

MS signs and symptoms	No sign present	Mild	Moderate	Severe	*p*
Sensory disturbances	24.24% (33)	33.33% (21)	20% (15)	37.5% (8)	.70
Motor deficit	22.22% (27)	30.95% (42)	50.00% (4)	0% (1)	.57
Spasticity of the lower limbs	21.62% (37)	29.41% (34)	75% (4)	0% (1)	.12
Balance problems	32% (25)	22.73% (44)	33.33% (6)	0% (1)	.71
Visual problems	27.03% (37)	26.09% (23)	29.41% (17)	‐	1.00
Bowel signs	22.72% (44)	22.72% (22)	62.5% (8)	33.33% (3)	.12
Bladder signs	30.77% (26)	27.78% (36)	21.42% (14)	0% (1)	.82
Speech problems (dysarthria)	21.05% (57)	47.37% (19)	‐	‐	.04

Correlations between general sexual performance along with its detailed aspects and demographic factors, MS symptoms, depression, and fatigue severity are presented in Table 4.

**Table 4:** MS parameters and FSFI general score with subscales

Feature	General FSFI	Desire	Arousal	Orgasm	Lubrication	Satisfaction	Pain
Age	−0.02 0.83	−0.26 0.02	−0.14 0.19	0.05 0.67	0.01 0.91	−0.10 0.35	0.26 0.02
Education level^a^	0.22 0.05	−0.05 0.68	0.02 0.83	0.16 0.15	0.23 0.04	0.00 0.99	0.23 0.04
Area of residence^b^	0.34 0.002	0.25 0.02	0.34 0.001	0.19 0.09	0.31 0.005	0.14 0.20	0.23 0.04
Self‐reported EDSS score^c^	−0.06 0.76	−0.21 0.28	−0.00 0.98	−0.16 0.43	0.07 0.71	−0.13 0.50	0.21 0.29
Age at diagnosis	−0.27 0.02	−0.40 <0.001	−0.33 0.003	−0.12 0.30	−0.09 0.41	−0.33 0.003	−0.01 0.91
Disease duration	0.22 0.06	0.07 0.55	0.11 0.33	0.16 0.16	0.10 0.40	0.23 0.04	0.27 0.02
PHQ‐9 score	−0.34 0.003	−0.23 0.03	−0.19 0.08	−0.26 0.02	−0.23 0.04	−0.22 0.04	−0.22 0.05
FSS score	−0.23 0.05	−0.27 0.01	−0.00 0.98	−0.16 0.43	−0.07 0.71	−0.13 0.50	−0.21 0.29
Sensory disturbances	−0.04 0.72	0.00 0.97	0.00 0.97	0.14 0.19	−0.15 0.18	0.09 0.41	0.09 0.40
Motor deficit	−0.24 0.04	−0.14 0.20	−0.21 0.06	−0.10 0.38	−0.31 0.01	−0.13 0.24	−0.02 0.89
Spasticity of the lower limbs	−0.27 0.02	−0.14 0.21	−0.23 0.04	−0.18 0.12	−0.24 0.03	−0.08 0.45	−0.12 0.27
Balance problems	−0.05 0.66	0.14 0.22	−0.05 0.67	−0.07 0.53	−0.10 0.38	0.08 0.50	−0.07 0.52
Visual problems	0.02 0.89	0.12 0.27	0.07 0.52	0.18 0.10	−0.14 0.21	0.10 0.39	−0.07 0.53
Bowel signs	−0.20 0.08	−0.18 0.09	−0.20 0.06	−0.16 0.16	−0.18 0.10	−0.11 0.34	−0.05 0.62
Bladder signs	−0.05 0.63	0.03 0.82	−0.08 0.49	−0.05 0.64	0.01 0.94	0.10 0.37	−0.03 0.77
Speech problems (dysarthria)	−0.20 0.08	−0.14 0.21	−0.25 0.03	−0.10 0.36	−0.19 0.09	0.00 0.99	−0.10 0.36

Cell organization: upper value–*R*; lower value–*p*; grey shade values‐ statistically significant.

a Codes applied with increasing level of education, beginning from primary/lack of education and ending on full higher education.

b Codes applied with increasing size of place of residence, beginning from village and ending on town with 100.000 or more inhabitants.

c Smaller subgroup; *N* (26‐29).

FSFI, female sexual function index; FSS, fatigue severity scale.

A negative correlation was observed between the general FSFI score and age at diagnosis (*r* = −.27; *p* < .02). Positive correlations were noticed between the general FSFI score and both education level (*r* = .22; *p* < .05) and area of residence (*r* = .34; *p* < .002). A weak correlation was observed between disease duration and the general FSFI score, without reaching statistical significance (*r* = .22; *p* < .06). There was no correlation between age and the general FSFI score, although opposing correlations were observed within its subscales: positive in pain subscale (*r* = .26; *p* < .02) and negative in desire subscale (*r* = −.26; *p* < .02).

General, self‐reported EDSS score was not associated with any aspect of sexuality analyzed in the presented study.

Increase in PHQ‐9 score had a negative association with the general FSFI score (*r* = −.34; *p* < .003) and almost all of its subscales, as presented in the Table 4. FSS score correlated negatively with FSFI score as well (−.23; *p* < .05).

Regarding parameters of MS, FSFI score was negatively correlated with motor deficit (*r* = −.24; *p* < .04) and lower limb spasticity (*r* = −.27; *p* < .02). The impact of bowel (*r* = −.20; *p* < .08) and speech problems (*r* = −.20; *p* < .08) was close to statistical significance. All detailed correlations between subscales are also presented in Table 4.

## 4 Discussion

Epidemiological data suggest that the prevalence of significant sexual dysfunction in general population oscillates around 12%, while in the analyzed group it was more than twice as high, reaching 27% (Shifren, Monz, Russo, Segreti, & Johannes, 2008). According to published studies, RRMS is a form of multiple sclerosis least frequently affected by SD (Mohammadi et al., 2013; Zivadinov et al., 1999). Other forms of the disease have a much higher proportion of sexual dysfunction, reaching up to 100% for the long lasting form of the disease (Zorzon et al., 1999). Due to the above, female patients with forms other than relapsing‐remitting multiple sclerosis are relatively easily identified as high‐risk patients for SD occurrence, whereas patients with RRMS are not so easily suspected of a such dysfunction. Furthermore, non‐relapsing‐remitting forms of the disease are usually associated with a worse clinical condition and higher EDSS scores (Tremlett, Paty, & Devonshire, 2006). In such patients sexual dysfunction may be often considered to be a symptom of less importance. RRMS is also the most common form of the disease and has the earliest onset (Ebers, 2001; Goodin, 2014). All of the reasons mentioned above may lead to the idea that the analysis of factors associated with SD in this very particular group of patients may result in achieving practical clinical benefits. These benefits may include easier identification of patients with higher risk of SD in female patients with RRMS, which happens to be the biggest subgroup in MS‐affected population (Ebers, 2001).

Demographic and psychosocial factors were the first group of data analyzed in the study. Most of the publications referring to sexuality in MS indicate several factors with negative impact on sexuality: increasing age (Darija et al., 2015; Merghati‐Khoei et al., 2013; Mohammadi et al., 2013; Zivadinov et al., 1999; ), low level of education (Zivadinov et al., 1999), unemployment (Mohammadi et al., 2013; Zivadinov et al., 1999; ), and lack of partner (Zivadinov et al., 1999). The presented study confirmed the association with low level of education in selective RRMS group but only in terms of sexual performance. There appears to be a negative relationship between the size of town of residence and both sexual performance and occurrence of sexual dysfunction. No data regarding this matter in a similar population was identified in the literature. The impact of the area of residence may be in fact a result of the socio‐economic status, which corresponds with the area of residence in Poland (rural residents are usually of lower socio‐economic status). An interesting phenomenon was observed‐ increasing age had a negative impact on desire, but a positive on pain during intercourse. As a result the association between age and the general FSFI score was not significant. The selected analyzed group does not seem to differ from the general MS population in terms of relationship of demographic parameters and sexuality (Darija et al., 2015; Merghati‐Khoei et al., 2013; Mohammadi et al., 2013; Zivadinov et al., 1999).

The second group of analyzed factors included characteristics of the underlying disease (MS) and its physical signs and symptoms. Some of the studies indicated negative association between MS duration and sexual performance (Merghati‐Khoei et al., 2013; Mohammadi et al., 2013), some did not report such an association (Darija et al., 2015; Ghajarzedeh et al., 2014; Zivadinov et al., 1999). However, the study of Merghati‐Khoei et al. (2013) performed on 132 women, demonstrated only weak correlation (*r* = .22) between length of the disease and general sexual performance. The study of Mohammadi et al. (2013) performed on a relatively big population (*n* = 226), indicated duration of the disease longer than 9 years as one of the strongest predictors of SD (aOR = 3.13). However, the population of the study of Mohammadi et al. (2013) included patients with all forms of the disease and higher proportion of SD (55.3%). There was no association between the duration of the disease and SD occurrence in our study. Furthermore, the longer the disease lasted, the better the scores were regarding satisfaction and pain subscales, which is an observation that had not been mentioned earlier in the literature. It is probable that patients with longer duration of the disease adapt to sexual problems and for this reason are more satisfied with intercourses in comparison to patients with a relatively short duration of the disease, as the latter suffer from a sudden deterioration of the quality of life. The higher score in pain subscale may be a result of a disturbed sensory response, which is more frequent in long lasting disease (Zackowski, Wang, McGready, Calabresi, & Newsome, 2015). The negative correlation between age at the onset of the disease and FSFI score is an interesting observation and contrary to the results found in literature. The previously mentioned study of Merghati‐Khoei et al. (2013) negated the existence of such relationship. On the other hand, the study of Zivadinov et al. (1999) performed on 70 women, revealed an opposite correlation. However, both studies used different tool to assess SD. Moreover, mentioned studies concerned all forms of MS including those beginning at an older age and lasting for a shorter period. The presented results might be justified by the fact that patients with earlier onset of the disease have less sexual experience before the disease and for this reason find their “after‐onset” sexual life to be better.

The studies of Zivadinov et al. (1999; 70 women) and Fraser et al. (2008; 219 women) observed negative associations between urinary problems and sexuality. The previously mentioned study of Zivadinov et al. (1999) revealed correlation between sphincter (bowel) problems and sexual problems. The study of Lew‐Starowicz and Rola (2014), performed on Polish population of 137 women, analyzed bladder and bowel dysfunction altogether but nevertheless observed significant association with sexuality. Our results did not confirm such a relationship in terms of bladder problems. It may be due to the fact that more severe forms of MS other than RRMS are associated with a higher incidence of sphincteric dysfunction. Speech disturbances (dysarthria) were the only MS sign associated with SD occurrence. The previously mentioned study of Lew‐Starowicz and Rola (2014) observed no association of brainstem involvement with sexuality. The brainstem problems may be considered as a similar group of signs. This relation may be justified by a particularly psychologically disabling effect of problems with speech, which have a significant impact on almost every aspect of life (Yorkston, Baylor, & Amtmann, 2014). The correlation between FSFI score and intensity of speech problems was not significant (*p* < .08) but there were only patients with no speech problems and with minor problems, as presented in Table 3, which could make possible correlation more difficult to obtain. Motor impairment, including lower limb spasticity, correlated with FSFI score, but not with SD occurrence. This exact correlation was also observed by Lew‐Starowicz and Rola (2014). The lack of association of motor deficit with SD occurrence in our study may lead to conclusion that any form of motor impairment has a relatively low impact on sexual performance. However, it is important to remember that the analyzed group consisted of patients without active relapse and the average EDSS score was low (2.03).

Depression and chronic fatigue syndrome presence/severity were the last parameters analyzed in the presented study. Depression is known to be one of the most common comorbidities in MS and remains a strong risk factor for SD in general population not affected by MS (Baldwin, 2001; Yorkston et al., 2014). Similarly, severe fatigue, a typical MS symptom, is associated with secondary SD (Lew‐Starowicz & Gianotten, 2015). A significant association was observed between PHQ‐9 score and SD occurrence in the presented results. In addition, PHQ‐9 score correlated negatively with FSFI general score and all but one of its subscales—a rate of correlations which was not observed in case of any other factor presented in our study. This relationship is widely confirmed in literature in all forms of MS (Ghajarzedeh et al., 2014; Lew‐Starowicz & Rola, 2014; Zivadinov et al., 1999). Fatigue severity in the analyzed population also correlated with general FSFI score, but did not present any association with SD occurrence. However, FSS score presented mild negative correlation with FSFI score, which may indicate the association with sexuality in this group. The correlation was of marginal significance, however, a more significant (*p* < .01) correlation of the specific desire subscale was observed. The overall impact of fatigue on sexuality may therefore lie in the lack of desire. The effect of fatigue on female sexuality understudied in comparison to depression, but its negative effect was previously confirmed in the literature (Zivadinov et al., 1999).

Obtained results suggest that female respondents with RRMS who suffered from SD according to FSFI score lived in smaller towns, were less educated and more depressed, had stronger problems with communication, but did not differ from respondents without SD in terms of any other physical signs. However, it is important to point out the specific characteristics of the analyzed group—all of the mentioned conclusions concern relatively young women with less advanced disease. In female patients with RRMS who are not severely disabled, primary and secondary SD may play lesser role in comparison to tertiary SD. For this reason, psychosocial factors seem to be an important cause of SD in female patients with RRMS. Furthermore, it should be emphasized that SD in women with RRMS do not necessarily have to be associated with the disease, but may exist separately. The association of SD with lower educational level and residency in smaller towns might support the latter implication. This thesis is also supported by various correlations in which (in addition to depression) social factors such as education level and place of residence presented impact on sexual performance.

The main limitation of the study is the fact that 189 out of 218 (86.70%) initially completed questionnaires were collected online. This source of data can be less reliable than distribution of paper surveys among patients as there is no evidence whether respondents truly suffered from MS. However, questionnaires were distributed only among members of the Polish Society of Multiple Sclerosis and the Urszula Jaworska Foundation which increased chances of accessing MS patients. The second limitation and source of possible bias in the study is full anonymity of the subjects which disallows verification of neurological signs and symptoms of MS as well as the EDSS score. In order to minimalize possible bias this study focused on self‐reported symptoms and signs.

## 5 Conclusion

Sexual dysfunction in women with relapsing‐remitting multiple sclerosis, who are not severely disabled, is mostly associated with psychosocial factors, not directly with MS symptoms and signs. Female patients with RRMS who are more depressed, present speech disturbances, are less educated and live in smaller towns, should be considered high‐risk for sexual dysfunction and more attention should be focused on their sexuality. Therefore, probable causes of sexual dysfunction should be more likely searched for in mental state of these patients.

## Conflict of Interest

None declared.
